# Long-term survival after epirubicin, cisplatin and fluorouracil for gastric cancer: results of a randomized trial

**DOI:** 10.1038/sj.bjc.6690350

**Published:** 1999-04

**Authors:** J S Waters, A Norman, D Cunningham, J H Scarffe, A Webb, P Harper, J K Joffe, M Mackean, J Mansi, M Leahy, A Hill, J Oates, S Rao, M Nicolson, T Hickish

**Affiliations:** Cancer Research Campaign (CRC) Section of Medicine and Gastrointestinal Unit, Royal Marsden Hospital and Institute of Cancer Research, Downs Road, Sutton, Surrey SM2 5PT, UK; CRC Department of Medical Oncology, Christie Hospital, Wilmslow Road, Manchester M20 4BX, UK; Department of Medical Oncology, Guys Hospital, St Thomas Street, London SE1 9RT, UK; Imperial Cancer Research Fund Cancer Medicine Research Unit, St James University Hospital, Beckett Street, Leeds LS9 7TF, UK; CRC Department of Medical Oncology, University of Glasgow, Alexander Stone Building, Garscube Estate, Switchback Road, Bearsden Glasgow G61 1BD, UK; Department of Medical Oncology, St Georges Hospital, Blackshaw Road, London SW17 0QT, UK

**Keywords:** cancer, chemotherapy, ECF, FAMTX, gastric, oesophagogastric

## Abstract

We report the final results of a prospectively randomized study that compared the combination of epirubicin, cisplatin and protracted venous infusion fluorouracil (5-FU) (ECF regimen) with the standard combination of 5-FU, doxorubicin and methotrexate (FAMTX) in previously untreated patients with advanced oesophagogastric cancer. Between 1992 and 1995, 274 patients with adenocarcinoma or undifferentiated carcinoma were randomized from eight oncology centres in the UK and analysed for response and survival. The overall response rate was 46% (95% confidence interval (CI), 37–55%) with ECF, and 21% (95% CI, 13–28%) with FAMTX (*P* = 0.00003). The median survival was 8.7 months with ECF and 6.1 months with FAMTX (*P* = 0.0005). The 2-year survival rates were 14% (95% CI, 8–20%) for the ECF arm, and 5% (95% CI, 2–10%) for the FAMTX arm (*P* = 0.03). Histologically complete surgical resection following chemotherapy was achieved in ten patients in the ECF arm (three pathological complete responses to chemotherapy) and three patients in the FAMTX arm (no pathological complete responses). The ECF regimen resulted in a response and survival advantage compared with FAMTX chemotherapy. The probability of long-term survival following surgical resection of residual disease is increased by this treatment. The high response rates seen with ECF support its use in the neoadjuvant setting. © 1999 Cancer Research Campaign


*British Journal of Cancer* (1999) **80**(1/2), 269–272
© 1999 Cancer Research Campaign
Article no. bjoc.1998.0350

### Long-term survival after epirubicin, cisplatin and fluorouracil for gastric cancer: results of a randomized trial

**JS Waters[1], A Norman[1], D Cunningham[1], JH Scarffe[2], A Webb[1], P Harper[3], JK Joffe[4], M Mackean[5], J Mansi[6], M Leahy[2], A Hill[1], J Oates[1], S Rao[1], M Nicolson[1] and T Hickish[1]**

[1]Cancer Research Campaign (CRC) Section of Medicine and Gastrointestinal Unit, Royal Marsden Hospital and Institute of Cancer Research, Downs Road, Sutton, Surrey SM2 5PT, UK; [2]CRC Department of Medical Oncology, Christie Hospital, Wilmslow Road, Manchester M20 4BX, UK; [3]Department of Medical Oncology, Guys Hospital, St Thomas Street, London SE1 9RT, UK; [4]Imperial Cancer Research Fund Cancer Medicine Research Unit, St James University Hospital, Beckett Street, Leeds LS9 7TF, UK; [5]CRC Department of Medical Oncology, University of Glasgow, Alexander Stone Building, Garscube Estate, Switchback Road, Bearsden, Glasgow G61 1BD, UK; [6]Department of Medical Oncology, St Georges Hospital, Blackshaw Road, London SW17 0QT, UK

**Summary** We report the final results of a prospectively randomized study that compared the combination of epirubicin, cisplatin and protracted venous infusion fluorouracil (5-FU) (ECF regimen) with the standard combination of 5-FU, doxorubicin and methotrexate (FAMTX) in previously untreated patients with advanced oesophagogastric cancer. Between 1992 and 1995, 274 patients with adenocarcinoma or undifferentiated carcinoma were randomized from eight oncology centres in the UK and analysed for response and survival. The overall response rate was 46% (95% confidence interval (CI), 37–55%) with ECF, and 21% (95% CI, 13–28%) with FAMTX ($P$ = 0.00003). The median survival was 8.7 months with ECF and 6.1 months with FAMTX ($P$ = 0.0005). The 2-year survival rates were 14% (95% CI, 8–20%) for the ECF arm, and 5% (95% CI, 2–10%) for the FAMTX arm ($P$ = 0.03). Histologically complete surgical resection following chemotherapy was achieved in ten patients in the ECF arm (three pathological complete responses to chemotherapy) and three patients in the FAMTX arm (no pathological complete responses). The ECF regimen resulted in a response and survival advantage compared with FAMTX chemotherapy. The probability of long-term survival following surgical resection of residual disease is increased by this treatment. The high response rates seen with ECF support its use in the neoadjuvant setting.

**Keywords:** cancer; chemotherapy; ECF; FAMTX; gastric; oesophagogastric

The value of combination chemotherapy in advanced oesophagogastric cancer has been clarified. Three randomized clinical trials have demonstrated the superiority of chemotherapy over best supportive care alone (Murad et al, 1993; Pyrhonen et al, 1995; Glimelius et al, 1997). However, the optimal regimen has not yet been established. The combination of 5-fluorouracil (5-FU), adriamycin and methotrexate (FAMTX) has been considered standard therapy, with superior response and survival rates compared with previous regimens (Wils et al, 1991; Kelsen et al, 1992). A combination of cisplatin, epirubicin, leucovorin and 5-FU (PELF) has also demonstrated impressive response rates in a randomized study (Cocconi et al, 1994). The regimen of epirubicin, cisplatin and 5-FU (ECF) was developed at the Royal Marsden Hospital (RMH) and first reported in 1991 (Cunningham et al, 1991). The three drugs in this regimen were selected on the basis of their single agent activity in upper gastrointestinal tract tumours (Beer et al, 1983; Cersosimo and Hong, 1986; Machover et al, 1986), and on the synergy demonstrated between 5-FU and cisplatin in preclinical models (Etienne et al, 1991). The 5-FU is delivered as a protracted infusion as this schedule has produced higher response rates with less myelotoxicity compared with bolus administration in patients with colorectal cancer (Lokich et al, 1989). Following the demonstration of response rates of 71% with moderate toxicity in a phase II study (Findlay et al, 1994), we undertook a multicentre randomized study comparing ECF with FAMTX in advanced oesophagogastric cancer. The initial results of this trial were reported in 1996 when recruitment was completed (Webb et al, 1997). At that stage, an advantage for ECF in response rate and survival was evident. However, median follow-up was only 6.1 months and only 75% of patients had died. We now present a final survival analysis with all patients followed up for 26.9 months or more (median 44 months), and 95% of patients having died.

*Received 27 August 1998*
*Revised 4 November 1998*
*Accepted 6 November 1998*

*Correspondence to:* D Cunningham

### METHODS

Our methods have been described previously (Webb et al, 1997). Briefly, patients with inoperable adenocarcinoma or undifferentiated carcinoma of the oesophagus, oesophagogastric junction, or stomach were randomized to receive ECF or FAMTX chemotherapy. ECF chemotherapy was administered through a central venous catheter placed in the subclavian vein. 5-FU was given as a continuous intravenous infusion at a dose of 200 mg $m^{-2}$ $day^{-1}$ for up to 6 months. Epirubicin (50 mg $m^{-2}$) and cisplatin (60 mg $m^{-2}$) were given every 3 weeks to a maximum of 8 cycles. FAMTX chemotherapy comprised methotrexate 1500 mg $m^{-2}$ and 5-FU 1500 mg $m^{-2}$ on day 1, and doxorubicin 30 mg $m^{-2}$ on day 15. Cycles were repeated every 28 days to a maximum of 24 weeks. Patients were followed up with clinical and symptomatic

**Table 1** Overall objective response rates

| Response | ECF (*n* = 121) No. | % | FAMTX (*n* = 116) No. | % | *P* |
|---|---|---|---|---|---|
| CR + PR | 56 | 46 | 24 | 21 | 0.00003 |
| CR | 10 | 8 | 2 | 2 | |
| PR | 46 | 38 | 22 | 19 | |
| SD | 25 | 21 | 24 | 21 | |
| PD | 23 | 19 | 43 | 37 | |
| Insufficient treatment | | | | | |
| Early death | 7 | | 17 | | |
| Toxic death | 1 | | 2 | | |
| Toxicity | 5 | | 6 | | |
| Patient request | 4 | | – | | |

CR, complete response; PR, partial response; SD, stable disease; PD, progressive disease.

assessment at each visit, and computerized tomography (CT) scan and endoscopy at 12 and 24 weeks from the start of treatment. Responses were classified according to World Health Organization (WHO) criteria (Miller et al, 1981). In addition, histological confirmation at endoscopy or surgery was required to satisfy the definition of complete remission. Statistical methods used in the design and analysis of this trial have been described previously (Webb et al, 1997). Tumour response rates in the two arms were compared using the $\chi^2$ test. Patient survival and failure-free survival was examined using the Kaplan–Meier product-limit method, and treatment arms were compared with the log-rank test. Participants gave written informed consent before they entered the study, which was approved by the individual institutes' Ethics Committee.

### RESULTS

Between July 1992 and June 1995, 274 patients (137 in each arm) were randomized from eight oncology centres in the UK. Eighteen patients were ineligible as described in our previous report (Webb et al, 1997), and were excluded from the analysis. Therefore, 256 patients (126 ECF and 130 FAMTX) were assessable on an intention-to-treat basis. The patient characteristics were well matched between treatment groups (Webb et al, 1997).

### Response

Response analysis is now complete and includes 237 evaluable patients (121 ECF and 116 FAMTX). A detailed analysis is presented in Table 1. The response rate was 46% (95% confidence interval (CI), 37–55%) in the ECF arm, and 21% (95% CI, 13–28%) in the FAMTX arm ($P$ = 0.00003). The response rate for locally advanced disease was 58% (95% CI, 43–72%) (eight complete responses and 17 partial responses) for ECF and 20% (95% CI, 9–34%) (two complete responses and seven partial responses) for FAMTX ($P$ = 0.0002). For metastatic disease, the response rate was 40% (95% CI, 29–52%) for ECF and 21% (95% CI, 13–33%) for FAMTX.

### Survival

All eligible patients were included in the survival analysis on an intention-to-treat basis. All surviving patients have now been followed up for at least 26.9 months (median 44 months), and 95% of patients have died. The median survival time was 8.7 months with ECF and 6.1 months with FAMTX ($P$ = 0.0005) (Figure 1). The 1- and 2-year survival rates were 37% (95% CI, 28–45%) and 14% (95% CI, 8–20%) for the ECF arm, and 22% (95% CI, 15–29%) and 5% (95% CI, 2–10%) for the FAMTX arm. The

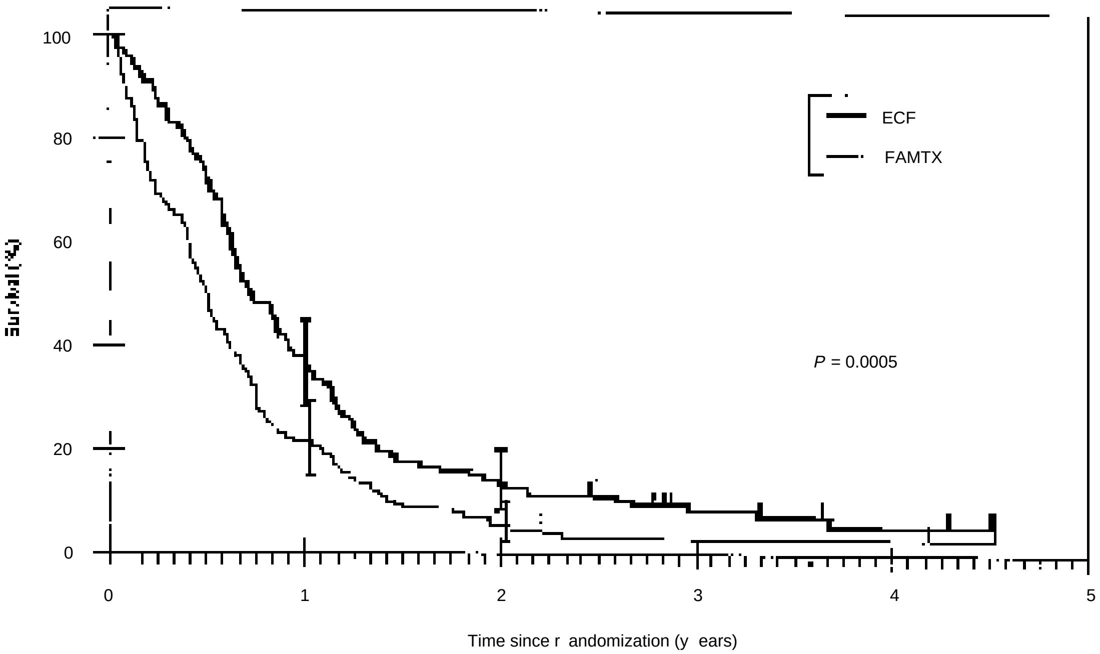

**Figure 1** Overall survival curve

© Cancer Research Campaign 1999

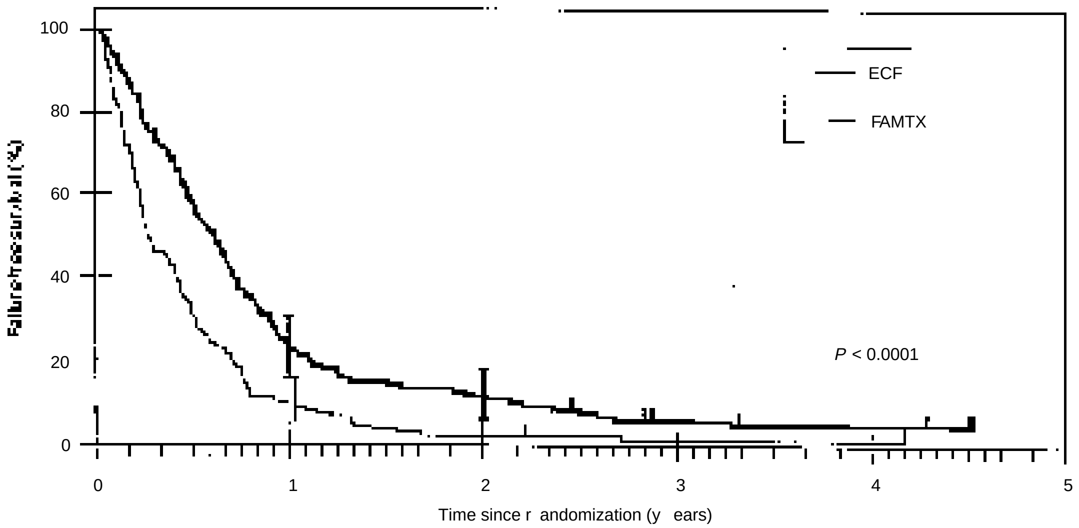

**Figure 2** Failure-free survival curve median failure-free survival time was 7.4 months with ECF and 3.3 months with FAMTX ($P < 0.0001$) (Figure 2).

### Surgery

Twenty-five patients have had a surgical procedure following chemotherapy (19 ECF and six FAMTX). Resection was not possible or the procedure was performed purely for palliation of symptoms in seven patients (six ECF and one FAMTX). Five patients had attempted resection of their tumour, but histological examination revealed that excision was incomplete (four ECF and one FAMTX). A histologically complete resection was performed in ten patients in the ECF arm, three of whom had a pathological complete response to chemotherapy. Four patients treated with FAMTX had a complete resection with no pathological complete responses.

### Long-term survival

Twenty-four patients have survived for 2 or more years (range 24.6–55.1 months) from randomization (17 (13.5%) ECF and seven (5.4%) FAMTX; $P = 0.03$). Thirteen patients in the ECF arm and three in the FAMTX arm had locally advanced disease. Potentially curative surgery was performed in nine of these patients following chemotherapy (seven ECF and two FAMTX), and consolidation radiotherapy performed in two (both ECF). A total of eight patients (five ECF and three FAMTX) have gone on to receive treatment with a variety of palliative chemotherapy regimens following progressive or recurrent disease, and six patients have received no further active treatment. Thirteen patients remain alive (26.9–55.1 months from randomization), of whom eight are free of disease: six patients in the ECF arm and one in the FAMTX arm who subsequently had a complete surgical resection, and one patient treated with ECF who had initially been referred for chemotherapy following an incomplete surgical resection and received no additional treatment subsequently.

### DISCUSSION

We have shown a definite advantage for the ECF regimen over FAMTX in the treatment of advanced oesophagogastric adenocarcinoma. This final analysis confirms and strengthens the conclusions of our initial analysis of this study (Webb et al, 1997), showing improved response rates and survival for the ECF arm, which are sustained beyond 2 years. High levels of activity have been demonstrated for a number of regimens in phase II studies (Preusser et al, 1989; Murad et al, 1993; Findlay et al, 1994), but randomized comparisons with established regimens have often shown little or no advantage for the new regimen (Kelsen et al, 1992; Wilke et al, 1995). The EORTC study comparing FAMTX with 5-FU, doxorubicin and mitomycin (FAM) (Wils et al, 1991) was the first study demonstrating a survival advantage between chemotherapy regimens, and FAMTX has remained the gold standard of therapy. Another recently reported phase II study has investigated a weekly regimen incorporating cisplatin, leucovorin-modulated 5-FU and epirubicin, with granulocyte colony-stimulating factor and glutathione support (Cascinu et al, 1997). A response rate of 62% was reported, with a median survival of 11 months. The response rate of the ECF regimen was 71% in the phase II study (Findlay et al, 1994). The lowering of the response rate in this phase III randomized trial is likely to be due to elimination of case-selection bias, a commonly observed phenomenon. ECF would therefore be an appropriate regimen to act as comparator in future trials.

Long-term survival in advanced oesophagogastric cancer is very rare. Previous studies have reported 2-year survival rates for the FAMTX regimen of 9–10% (Wils et al, 1991; Murad et al, 1993). In our study, ECF produced a 2-year survival rate of 13.5% compared with 5.4% for FAMTX. It is notable that ECF chemotherapy resulted in tumour downstaging sufficient to allow complete resection of previously inoperable tumours in ten patients, three of whom had no residual tumour in the resected specimen. Six of these patients remain disease-free. In contrast, only four patients treated with FAMTX had complete resections performed, only one of whom is currently disease-free.

*© Cancer Research Campaign 1999*

The results of surgery alone in oesophagogastric cancer are poor. Five-year survival rates of 21% have been reported following curative resection (Allum et al, 1989). The high response rates seen with ECF, particularly in patients with locally advanced disease, and the extended survival of patients going on to have complete surgical excision of residual disease provide support for the use of ECF in neoadjuvant therapy. Surgeons should be reassured by the low rates of progressive disease with this regimen. In this trial, only 5% of patients with locally advanced disease progressed while on ECF chemotherapy and are likely to represent a poor prognostic group. Rather than delaying surgery, neoadjuvant ECF may in fact render tumours more easily resectable, allowing more curative procedures to be undertaken. A number of phase II studies have investigated preoperative chemotherapy with a variety of regimens in oesophagogastric cancer, both in patients with resectable and non-resectable disease (Wilke et al, 1989; Plukker et al, 1991, 1995; Rougier et al, 1994; Melcher et al, 1996). These support the principle of neoadjuvant chemotherapy, producing extended disease-free and overall survival compared with historical controls. High rates of complete resection have been reported in patients with initially unresectable locally advanced disease. These series are difficult to compare because of different criteria for selecting patients, and the small numbers of patients involved. The MAGIC trial, which randomizes patients with operable gastric cancer to receive peri-operative ECF or surgery alone, currently being undertaken by the Medical Research Council in conjunction with the British Gastric Cancer Group, will provide definitive evidence as to the efficacy of this approach.

### ACKNOWLEDGEMENTS

We thank Michael Crawford for his contribution to this study.

### REFERENCES

Allum WH, Powell DJ, McConkey CC and Fielding JW (1989) Gastric cancer: a 25-year review. *Br J Surg* **76**: 535–540

Beer M, Cocconi G, Ceci G, Varini M and Cavalli F (1983) A phase II study of cisplatin in advanced gastric cancer. *Eur J Cancer Clin Oncol* **19**: 717–720

Cascinu S, Labianca R, Alessandroni P, Marcellini M, Silva RR, Pancera G, Testa E, Martignoni G, Barni S, Frontini L, Zaniboni A, Luporini G, Cellerino R and Catalano G (1997) Intensive weekly chemotherapy for advanced gastric cancer using fluorouracil, cisplatin, epi-doxorubicin, 6S-leucovorin, glutathione, and filgrastim: a report from the Italian Group for the Study of Digestive Tract Cancer. *J Clin Oncol* **15**: 3313–3319

Cersosimo RJ and Hong WK (1986) Epirubicin: a review of the pharmacology, clinical activity, and adverse effects of an adriamycin analogue. *J Clin Oncol* **4**: 425–439

Cocconi G, Bella M, Zironi S, Algeri R, Di Costanzo F, De Lisi V, Luppi G, Mazzocchi B, Rodino C, Soldani M, Gilli G and Finardi C (1994) Fluorouracil, doxorubicin, and mitomycin combination versus PELF chemotherapy in advanced gastric cancer: a prospective randomized trial of the Italian Oncology Group for Clinical Research. *J Clin Oncol* **12**: 2687–2693

Cunningham D, Mansi J, Ford HT, Nash AT and Menzies Gow N (1991) Epirubicin, Cisplatin and 5-Fluorouracil (ECF) Is Highly Effective In Advanced Gastric Cancer (Meeting Abstract). *Proc Annu Meet Am Soc Clin Oncol* **10**: 136

Etienne MC, Bernard S, Fischel JL, Formento P, Gioanni J, Santini J, Demard F, Schneider M and Milano G (1991) Dose reduction without loss of efficacy for 5-flourouracil and cisplatin combined with folinic acid. In vitro study on human head and neck carcinoma cell lines. *Br J Cancer* **63**: 372–377

Findlay M, Cunningham D, Norman A, Mansi J, Nicolson M, Hickish T, Nicolson V, Nash A, Sacks N, Ford H, Carter R and Hill A (1994) A phase II study in advanced gastro-esophageal cancer using epirubicin and cisplatin in combination with continuous infusion 5-fluorouracil (ECF). *Ann Oncol* **5**: 609–616

Glimelius B, Ekstrom K, Hoffman K, Graf W, Sjoden PO, Haglund U, Svensson C, Enander LK, Linne T, Sellstrom H and Heuman R (1997) Randomized comparison between chemotherapy plus best supportive care with best supportive care in advanced gastric cancer. *Ann Oncol* **8**: 163–168

Kelsen D, Atiq OT, Saltz L, Niedzwiecki D, Ginn D, Chapman D, Heelan R, Lightdale C, Vinciguerra V and Brennan M (1992) FAMTX versus etoposide, doxorubicin, and cisplatin: a random assignment trial in gastric cancer. *J Clin Oncol* **10**: 541–548

Lokich JJ, Ahlgren JD, Gullo JJ, Philips JA and Fryer JG (1989) A prospective randomized comparison of continuous infusion fluorouracil with a conventional bolus schedule in metastatic colorectal carcinoma: a Mid-Atlantic Oncology Program Study. *J Clin Oncol* **7**: 425–432

Machover D, Goldschmidt E, Chollet P, Metzger G, Zittoun J, Marquet J, Vandenbulcke JM, Misset JL, Schwarzenberg L, Fourtillan JB, Gaget H and Mathe G (1986) Treatment of advanced colorectal and gastric adenocarcinomas with 5-fluorouracil and high-dose folinic acid. *J Clin Oncol* **4**: 685–696

Melcher AA, Mort D and Maughan TS (1996) Epirubicin, cisplatin and continuous infusion 5-fluorouracil (ECF) as neoadjuvant chemotherapy in gastro-oesophageal cancer. *Br J Cancer* **74**: 1651–1654

Miller AB, Hoogstraten B, Staquet M and Winkler A (1981) Reporting results of cancer treatment. *Cancer* **47**: 207–214

Murad AM, Santiago FF, Petroianu A, Rocha PR, Rodrigues MA and Rausch M (1993) Modified therapy with 5-fluorouracil, doxorubicin, and methotrexate in advanced gastric cancer. *Cancer* **72**: 37–41

Plukker JT, Mulder NH, Sleijfer DT, Grond J and Verschueren RCJ (1991) Chemotherapy and surgery for locally advanced cancer of the cardia and fundus: phase II study with methotrexate and 5-fluorouracil. *Br J Surg* **78**: 955–958

Plukker JT, Sleijfer DT, Verschueren RCJ, Van der Graaf WTA and Mulder NH (1995) Neo-adjuvant chemotherapy with carboplatin, 4-epiadriamycin and teniposide (CET) in locally advanced cancer of the cardia and lower oesophagus: a phase II study. *Anticancer Res* **15**: 2357–2362

Preusser P, Wilke H, Achterrath W, Fink U, Lenaz L, Heinicke A, Meyer J, Meyer HJ and Buente H (1989) Phase II study with the combination etoposide, doxorubicin, and cisplatin in advanced measurable gastric cancer. *J Clin Oncol* **7**: 1310–1317

Pyrhonen S, Kuitunen T, Nyandoto P and Kouri M (1995) Randomised comparison of fluorouracil, epidoxorubicin and methotrexate (FEMTX) plus supportive care with supportive care alone in patients with non-resectable gastric cancer. *Br J Cancer* **71**: 587–591

Rougier P, Mahjoubi M, Lasser P, Ducreux M, Oliveira J, Ychou M, Pignon JP, Elias D, Bellefqih S, Bognel C, Lusinchi A, Cvitkovic E and Droz JP (1994) Neoadjuvant chemotherapy in locally advanced gastric carcinoma – a phase II trial with combined continuous intravenous 5-fluorouracil and bolus cisplatinum. *Eur J Cancer* **30A**: 1269–1275

Webb A, Cunningham D, Scarffe JH, Harper P, Norman A, Joffe JK, Hughes M, Mansi J, Findlay M, Hill A, Oates J, Nicolson M, Hickish T, O'Brien M, Iveson T, Watson M, Underhill C, Wardley A and Meehan M (1997) Randomized trial comparing epirubicin, cisplatin, and fluorouracil versus fluorouracil, doxorubicin, and methotrexate in advanced esophagogastric cancer. *J Clin Oncol* **15**: 261–267

Wilke H, Preusser P, Fink U, Gunzer U, Meyer HJ, Meyer J, Siewert JR, Achterrath W, Lenaz L, Knipp H and Schmoll HJ (1989) Preoperative chemotherapy in locally advanced and nonresectable gastric cancer: a phase II study with etoposide, doxorubicin and cisplatin. *J Clin Oncol* **7**: 1318–1326

Wilke H, Wils J, Rougier P, Lacave A, Van Cutsem E, Vanhoeferi U, Sahmoud T, Curran D and Marinus A (1995) Preliminary analysis of a randomized phase III trial of FAMTX versus ELF versus cisplatin/FU in advanced gastric cancer. A trial of the EORTC Gastrointestinal Tract Cancer Cooperative Group and the AIO (Arbeitsgemeinschaft Internistische Onkologie) (Meeting abstract). *Proc Annu Meet Am Soc Clin Oncol* **14**: 206

Wils JA, Klein HO, Wagener DJ, Bleiberg H, Reis H, Korsten F, Conroy T, Fickers M, Leyvraz S, Buyse M and Duez N (1991) Sequential high-dose methotrexate and fluorouracil combined with doxorubicin – a step ahead in the treatment of advanced gastric cancer: a trial of the European Organization for Research and Treatment of Cancer Gastrointestinal Tract Cooperative Group. *J Clin Oncol* **9**: 827–831

© Cancer Research Campaign 1999
